# Perioperative Communication Between Nurse Anesthetists and Patients: A 30-Year Experience From Sweden

**DOI:** 10.7759/cureus.80108

**Published:** 2025-03-05

**Authors:** Ferid Krupić, Melissa Krupić, Edna Supur, Jasmin Alić, Edin Ališić

**Affiliations:** 1 Department of Anaesthesiology, Institute of Clinical Sciences, Sahlgrenska Academy, University of Gothenburg, Gothenburg, SWE; 2 Department of Gynaecology, Sahlgrenska University Hospital-Östra, Gothenburg, SWE; 3 Paediatrics, School of Medicine University of Sarajevo (MFUNSA), Sarajevo, BIH; 4 Urology Clinic, Clinical Center University of Sarajevo, Sarajevo, BIH; 5 Department of Anaesthesiology, Sahlgrenska University Hospital-Östra, Gothenburg, SWE

**Keywords:** communication, nurse anaesthetist, perioperative dialogue, qualitative research, sweden

## Abstract

Introduction

Nurse anesthetists (NAs) rely on various tools to perform their daily tasks effectively, with communication being one of the most essential during the perioperative phase. The study aimed to explore NAs' experiences with the perioperative dialogue with patients and how this dialogue has evolved over the past 30 years.

Materials and methods

The study employed a qualitative design, with data gathered through three group interviews focusing on NAs' experiences. Interpretive content analysis, following the approach of Graneheim and Lundman, was used. Initially, 27 NAs were recruited, and 18 (three men and 15 women) participated in the interviews. Their ages ranged from 33 to 72 years, with work experience spanning 17 to 42 years.

Results

The text analysis identified three categories: advantages of perioperative dialogue, disadvantages of its absence, and suggestions for improvement. Key challenges included maintaining continuity of care, ensuring a high level of patient and NA safety, reducing care-related complications, minimising patient socialisation, providing incomplete care, and increasing stress for both NAs and patients. The NAs also offered several suggestions for improvement.

Conclusion

Perioperative meetings should be better structured to improve communication and assess outcomes. Enhancing patient involvement, developing NAs' skills, and providing clearer information in multiple languages could improve satisfaction and safety. Further research is needed to establish the dialogue’s role as a guiding principle for staff and patients.

## Introduction

To become a registered nurse anesthetist (NA) in Sweden, a nurse must complete a one-year specialized training program in anesthetic nursing. NAs are responsible for all aspects of anesthesia but work under the direct or indirect supervision of an anesthetist, following their established care plan [1]. Their work is defined by respect, responsibility, and openness, requiring them to be attuned to the patient’s condition and the perioperative encounter. This encounter aims to establish effective communication, ensuring patient safety [2].

Effective communication is one of the key tools NAs use in their daily practice, particularly during the perioperative phase of surgery. The term “perioperative” refers to the period surrounding surgery, encompassing pre-, intra-, and postoperative care provided by both anesthetists and NAs [3]. The perioperative dialogue allows NAs to interact with patients before and during anesthesia, fostering trust and rapport. Through these interactions, the NA assumes responsibility for the patient’s care, while the patient places their well-being in the hands of the NA [1-3].

This dialogue helps NAs understand patients’ experiences, alleviating preoperative distress such as anxiety, fear, or depression [4]. Continuity in communication becomes an ethical act, demonstrating the NA’s commitment to patient care [3]. Patients often feel reassured knowing that an NA is consistently present throughout the perioperative process [2-4]. However, this requires the NA to have the willingness, energy, and capacity to integrate this responsibility alongside their other clinical duties [5].

For NAs, maintaining dialogue continuity not only enhances patient care but also creates a shared experience between the NA and the patient [6]. This shared connection fosters a sense of togetherness, providing a solid foundation for the patient’s recovery and post-surgical well-being [1,7]. Conversely, the absence of this connection may lead to patient suffering, with negative consequences such as inadequate information, exclusion from decision-making, and a lack of involvement in the care process [4]. Active participation in decision-making is crucial for high-quality patient care [2,4]. The NA’s presence and patient-centered approach ensure that each patient is recognized as an individual within the caregiving process [5,8-10]. Perioperative dialogue is fundamental in helping patients feel safe and supported in an unfamiliar and sometimes overwhelming medical environment. Meeting the NA before surgery can enhance this sense of security, as familiarity with the caregiver can ease the transition into the operating room [11,12]. The theoretical foundation of perioperative care is rooted in caritative care theory, which emphasizes humanity, compassion, and ethical responsibility based on human dignity [11]. This theory incorporates a humanistic perspective, respect, and humility toward the individual, further developing into three key principles: courage (the nurse’s commitment to being present for the patient), guilt (the nurse’s responsibility to act as the patient’s protector), and trust (the patient’s reliance on the nurse for care and support). Perioperative care theory emphasizes a holistic approach, recognizing the interconnectedness of body and soul. The nurse’s attitude, sense of responsibility, and commitment to building a therapeutic relationship are all established from the very first dialogue with the patient [11,13,14].

The perioperative dialogue fosters a sense of safety for patients in the operating room. It requires NAs to recognize patients' inherent strengths and support them in the path they choose when entrusting their lives to the NA. This dialogue helps safeguard patients' dignity by empowering healthcare professionals in their care roles. Further research is needed to better understand the dialogue’s significance as a guiding principle for both patients and healthcare providers.

The study aimed to explore NAs' experiences with the perioperative dialogue with patients and how this dialogue has evolved over the past 30 years.

## Materials and methods

Design and participants

This study employed a qualitative design and was conducted in three departments - Orthopaedics, Gynaecology, and Obstetrics - across two hospitals in western Sweden (Sahlgrenska University Hospital Molndal and Östra). NAs were invited to participate voluntarily through direct contact from the author, and those who agreed received written information about the study. After meeting the participants, the study’s aim was presented. NAs were invited to participate voluntarily through direct contact from the authors, and those who agreed received written information about the study. After meeting the participants, the study’s aim was presented.

NAs with at least 15 years of experience and familiarity with the perioperative dialogue were selected, as their longer experience provided more opportunities to apply this approach. Of the 27 NAs initially recruited, 18 (three men and 15 women) participated. Their ages ranged from 33 to 72 years (median 55), with 17 to 42 years of work experience (median 29.5).

Data were collected through three group interviews, each consisting of six participants. The sample size was calculated using the formula presented by Marshall et al. (2013) [15].

 n = [ Z^2^xPx(1-P)] /D2

where Z - confidence level (1.96 for 95%), P - expected occurrence of the phenomenon, and D - desired precision.

Data collection

The author conducted data collection through group interviews using individualized open-ended questions, guided by an interview framework inspired by Kvale [15]. Between November 2022 and February 2023, three group interviews were held, each consisting of six NAs.

The interviews began with the question: "Could you please describe your experience of using the perioperative dialogue in your daily work?" The participants were encouraged to speak freely, using their own words, and to elaborate on their experiences as thoroughly as possible. The interviewer intervened only to ask follow-up questions or clarify responses.

The interviews took place in the orthopaedic and gynaecological wards, lasting between 42 and 75 minutes. All sessions were audiotaped and transcribed verbatim for analysis.

Analysis

A qualitative content analysis approach, following the method outlined by Graneheim and Lundman [16], was used to analyze and interpret the data. The analysis was conducted systematically in several stages.

First, the transcribed interview texts were read multiple times to gain a comprehensive understanding of the material. Words and sentences relevant to the study’s research questions and objectives were identified as meaning-bearing units. These units were then condensed - shortened while preserving their core meaning - to facilitate analysis.

Next, the condensed text was abstracted to a higher level by assigning codes, each representing a brief description of a sentence unit’s content. Codes with similar meanings were grouped into categories, organizing the data thematically. Finally, a central theme emerged by identifying the underlying meaning that connected multiple categories [16].

The results are presented with direct quotes from the interviews to illustrate key findings.

Ethics statement

As the study did not involve physical interventions or the collection of personal health information, ethical board approval was not required under Swedish law (Swedish Health Care Act) [17]. The study was conducted in strict accordance with the World Medical Association Declaration of Helsinki [18]. To ensure confidentiality, the identities of healthcare professionals were protected, with no names or personal identification numbers included in the article or any related publications.

## Results

Initially, 27 NAs were recruited, and 18 (three men and 15 women) participated in the interviews. Their ages ranged from 33 to 72 years, with a median age of 55 years. Their professional experience as NAs varied between 17 and 42 years, with a median of 29.5 years. Data were collected through three group interviews, each consisting of six participants. The demographic characteristics of the participants are summarized in Table 1.

**Table 1:** Characteristics of ealthcare professionals

Sex	Number
Male	3
Female	15
Age (years)	
30-40	3
41-50	6
51-60	7
61-70	1
>70	1
Workplace	
Orhopaedics	7
Gynecology/Obtertics	11
Experiences (years)	
10-20	5
21-30	4
31-40	6
41-50	3
Total	18

The analysis identified one overarching theme - the importance of perioperative dialogue over the past three decades - along with three main categories and eight subcategories, based on NAs' experiences of using perioperative dialogue in daily practice. The three main categories were advantages of using perioperative dialogue, disadvantages of its absence, and suggestions for improvement. A detailed overview of these categories and their subcategories is presented in Table 2.

**Table 2:** Overview of the theme, categories, and subcategories

Categories	Subcategories		Theme
Advantages of using the perioperative dialogue	Having continuity of care		
	Safety for the patient and healthcare professionals		
	Reducing complications in care		The importance of using perioperative dialogue
Disadvantages to the absence	Missing socialising with their patient		
	Not performing complete care for the patient		
	Causing increased stress for the patient and healthcare professionals		
Suggestions for improvement	Increasing staff strength		
	Hiring more educated managers		

Advantages of using perioperative dialogue

All interviewees expressed enthusiasm when sharing their experiences with perioperative dialogue. They unanimously reported that incorporating this dialogue throughout the nursing process contributed to continuity of care, beginning with the initial meeting the day before surgery. This interaction allowed patients to receive information, ask questions, and establish a sense of trust with their assigned NA.

On the day of surgery, this prior communication minimized the need for repeated explanations, as patients already had a clear understanding of the procedure. The perioperative dialogue also enhanced patient and NA safety, reduced perioperative and postoperative complications, and fostered a smoother surgical process.

Having continuity of care

The majority of NAs described the perioperative dialogue as a valuable tool for maintaining control over the situation before, during, and after surgery. Key factors enabling this were adequate staffing, good teamwork, effective organization, and supportive management. NAs highlighted the importance of having time to meet with both staff and patients, engage in continuous dialogue, read and familiarize themselves with patient information, oversee the surgical process, and follow up with patients in the postoperative ward. This continuity of care created a positive experience, with many NAs expressing feelings of fulfillment and energy after successfully completing their tasks.

One NA shared, “I informed my patient about the entire operation process, answered her questions, described everything that was waiting in the operating room, and assured her that I would be with her the entire time.” Another reflected on the emotional impact of this approach: “When my patient saw me the next day in surgery, she started crying with happiness.” The long-term connection was evident as one NA added, “I am still friends with my patient… 20 years after her operation.”

Safety for the patient and healthcare professionals

The continuity of care, coupled with clear communication between the NA, anaesthesiologists, and surgeons, along with patient preparation, was seen by all NAs as crucial in ensuring safety for both the patient and healthcare professionals. Having adequate time allowed NAs to respond swiftly in the event of unexpected situations, which further enhanced safety for both the patient and the healthcare team.

One NA commented, “I read about my patient, discussed the whole process with my doctor, and had good staff in the room… everything was under control.” Another expressed the sense of satisfaction derived from this approach: “Having a satisfied, safe patient and being satisfied with the day feels like dancing instead of working… absolutely fantastic.” Finally, one NA reflected on the ongoing monitoring in the postoperative ward: “On the postoperative ward, I see that everything is OK… what else do I need?”

Reducing complications in care

The majority of NAs highlighted the value of perioperative dialogue as an opportunity to address various patient concerns, including those who were anxious, nervous, or scared. By taking the time to communicate, NAs could prevent and address potential complications during the perioperative process. Being fully informed about the patient's condition and managing the situation in a professional manner contributed to a smoother process and better outcomes.

One NA shared, “I repeated the whole operation to my patient several times… she was so scared… the next day she came to the operation laughing.” Another recalled a particularly anxious patient: “I had an aurora (anxious?) patient… she was afraid of everything… but I helped her, and everything went very well.” Another participant noted, “A well-informed patient has no complications.”

Disadvantages of the absence of perioperative dialogue

However, not all NAs could consistently perform the perioperative dialogue. As healthcare practices evolved, there was a shift in the way patient interactions were conducted, making it more challenging for NAs to engage with their patients in the same way. In particular, meeting patients only on the day of surgery, rarely having time to read about them beforehand, and not being able to check in with patients after their operations led to a lack of socialization, incomplete care, and increased stress for both patients and NAs.

Missing socialization with patients

Many NAs described how the absence of perioperative dialogue often resulted in a rushed, impersonal experience. The interaction with patients was usually brief, taking place in corridors or moments before the operation. NAs had little time to familiarize themselves with their patients or to establish a connection.

One NA expressed, “Sometimes, it feels like we're working in a factory and not with people.” Another said, “A long time ago, I had time to read about my patients, but I don't have the same ability now.”

Not performing complete care for the patient

Without time for perioperative dialogue, NAs felt they could not offer comprehensive care. This lack of continuity left many NAs feeling dissatisfied with the care they were providing.

One participant noted, “I know that my patient was fine during the operation, but, what happened next, God only knows.” Another remarked, “During one and the same operation, I met three different patients… talk about continuity.”

Causing increased stress for the patient and healthcare professionals

Not being able to maintain continuous care for patients or to follow a routine contributed to heightened stress for both patients and NAs. The pressures of managing multiple tasks at once and a constantly shifting schedule were noted by several NAs.

One NA said, “With a stressed patient, everything takes twice as long compared with a non-stressed patient.” Another shared, “Sometimes, I have a younger colleague with me. I am in the postoperative ward and also have the viewfinder on me.”

Suggestions for improvement

The NAs in this study felt that the Swedish healthcare system's failure to recognize and address staffing shortages in a timely manner was a key factor in the current challenges. Many of those who had been employed for decades in the healthcare system retired, leaving a gap in expertise and leadership. NAs perceived this oversight as irresponsible and unacceptable. As a result, staff shortages and a decline in the quality of care persisted, worsened by the increasing number of unqualified managers.

The NAs strongly felt that leadership improvements and staff retention strategies were critical for addressing these challenges and improving the quality of patient care.

Increasing staff strength

All the NAs in this study expressed dissatisfaction with the current personnel policies, which they described as inadequate. The issue of understaffing - either due to a complete lack of staff or reliance on temporary or hired workers - was considered unacceptable by all the participants. This situation made it increasingly difficult to carry out a perioperative dialogue with patients, as the necessary time and resources were not available.

One NA stated, “There have to be more of us; otherwise, it is not possible to work in this way.” Another added, “A hired person wants and works for money, not to reintroduce the perioperative dialogue.”

Hiring more educated managers

The situation regarding managerial staff was equally troubling for the NAs. According to the majority of the participants, very few managers had received proper managerial training, and those who did often only received training once they had already assumed their roles. This lack of preparedness was viewed as highly unprofessional and ineffective in leading teams.

One NA commented, “Regarding my boss, it took a long time for him to become a real boss.” Another shared, “Every time I had a question and looked for my manager, she was at training.”

## Discussion

The findings of this study highlight a significant shift in the use and impact of perioperative dialogue in healthcare. In the past, NAs experienced a highly beneficial situation where they could meet their patients the day before surgery, engage in thorough discussions, and provide continuous care, which in turn contributed to increased safety and reduced complications. This continuity was seen as a crucial part of the process, fostering a sense of security for both the patient and the NA. However, as the study moves into the present, this ideal scenario has been disrupted. The absence of perioperative dialogue has resulted in reduced social interaction with patients, leading to a sense of incomplete care. This, in turn, has contributed to unnecessary stress for both the patients and the NAs. The NAs in the study expressed their concern that the lack of communication and personal engagement with patients had a negative impact on the quality of care provided.

Drawing on Eriksson’s perspective, which emphasizes that the mission of caring is to alleviate human suffering and serve life and health [12], the perioperative dialogue is an essential tool in this process. Through meaningful communication, the NA helps to satisfy the patient’s needs, providing space for questions and reassurance. This interaction not only ensures the patient’s understanding and comfort but also confirms their humanity, which is fundamental to the caregiving process [12,13]. It is this human connection that contributes to the holistic approach to care, where patients are not just seen as medical cases but as individuals in need of empathy and understanding. The lack of this crucial dialogue in recent times is seen as a detriment to the quality of care and the overall well-being of both patients and healthcare professionals.

The idea that the nurse plays a crucial role in confirming and establishing a caring relationship with the patient is highlighted here, and this process enables the patient to actively engage in their own care. With the perioperative dialogue, the nurse gains a deeper understanding of the patient's experiences, allowing them to better address any fears and alleviate suffering. This aligns with Eriksson’s notion of caring, which emphasizes the alleviation of suffering and the creation of a pathway for a meaningful relationship between the caregiver and patient [14].

This theme is reinforced by studies indicating that patients undergoing major surgeries, like hip or knee replacements, view these procedures as significant life events, and the nurses' awareness and communication have been shown to significantly reduce patients' anxiety and fears [19,20]. The NAs in this study, when able to use the perioperative dialogue, reported a sense of control and satisfaction in providing continuous care, ensuring patient safety, and preventing complications. This seamless experience was something that the NAs "boasted" about, as it led to positive outcomes for both the patient and healthcare professionals. However, the study suggests that in an ideal healthcare system with trained managers, sufficient staff, and appropriate resources, this level of care should be standard practice. In such a system, the use of perioperative dialogue would be commonplace, leading to better patient outcomes and a smoother healthcare process. Unfortunately, the current reality in Swedish healthcare, as described by the NAs, shows that time constraints, understaffing, and poor organization make it challenging to implement this practice consistently [10,21,22].

In the period when NAs had the opportunity to conduct a perioperative dialogue, they all described the experience positively, emphasizing the benefits of meeting patients the day before surgery, ensuring continuity of care, and enhancing safety for both themselves and their patients. This situation, while highlighted in the study, is not groundbreaking - it reflects the ideal functioning of a healthcare system with trained managers, sufficient staff, and adequate time for patient care. In a system like this, such practices should be the norm, as seen in other studies where patients undergoing hip replacement surgery reported excellent continuity of care due to the presence of their dedicated doctor and nurse throughout the process [23-26].

In these optimal work conditions, perioperative dialogue is crucial for maintaining control over the situation, ensuring the patient’s comfort, and addressing complications such as anxiety and pain. However, the study also reveals a gap between the ideal and the reality in today’s Swedish healthcare system. Despite the NAs' enthusiasm for using the perioperative dialogue, time constraints, staffing shortages, and poor organizational support make it difficult to carry out these practices consistently.

The results echo findings from a previous study by Hansson and Söderhamn (2004), which noted that NAs were more favorable toward using the perioperative dialogue compared to operating room nurses [27]. However, today's healthcare environment, burdened by a lack of resources and proper organization, has led to NAs not having the necessary time to perform the dialogue, engage with patients fully, and deliver quality care. This results in increased stress for both healthcare professionals and patients [27,28]. The lack of time and staff significantly impacts the ability to maintain the quality of care, which ultimately compromises the patient experience and healthcare outcomes [28].

The findings from the study highlight a critical issue in the current Swedish healthcare system: the inability to consistently perform the perioperative dialogue due to severe staff shortages and insufficiently trained managers. This situation has been exacerbated by a generational shift where many experienced staff members retired, but there weren’t enough new recruits to replace them. The gap in staffing has resulted in increased patient numbers and unchanged responsibilities, which places considerable strain on the remaining staff. Moreover, the lack of preparation for this shift in the workforce has caused a mismatch between the needs of the healthcare system and the available personnel [29, 30].

The government’s approach to hiring temporary staff has only added to the issue. Temporary staff, who are hired for a salary of SEK 100,000 (about 9,000 euros), do not hold any responsibility within the department, and in cases of extra work, they are not asked to stay. This situation has led to dissatisfaction among permanent state employees, who feel that this approach undermines their roles and increases the strain on the system, especially as the overall cost to the state has risen significantly.

In this environment, anaesthesiologists are the ones who, to some extent, still manage to conduct a form of perioperative dialogue when they meet with patients preoperatively. However, this dialogue is limited in quality, as doctors are generally not trained in the aspects of nursing and patient communication specific to the perioperative model. Furthermore, due to staff shortages, operating theatres are increasingly staffed by doctors rather than NAs, raising concerns about the future viability of the NA profession itself. The combination of inadequate training, staffing issues, and shifting roles within the healthcare system raises questions about the sustainability of the perioperative dialogue model and the long-term stability of the healthcare workforce in Sweden.

Implications for clinical practice

Our study highlights key insights relevant to clinical practice. In a genuine perioperative meeting, the patient is recognized as an active participant in shaping their identity and must remain at the center of all surgical events. However, interviews with NAs reveal both advantages and disadvantages of these meetings. As modern technology increasingly takes over, the patient’s individuality and integrity risk being overshadowed. To enhance patient-centered care, the healthcare system must address several areas: providing clear, detailed information at hospital admission, offering information in multiple languages, and ensuring patients feel valued and central to their care journey. Addressing these needs lays a strong foundation for improving care for both NAs and future patients.

Strengths and limitations of the study

Our study captures both the benefits and challenges of the brief perioperative meetings between NAs and patients over the past thirty years. A key strength is its clear depiction of changes in Swedish healthcare over time, reflecting generational shifts and varied responses to these changes. In addition, the study draws on the experiences of 18 NAs across three different clinics, offering a broad perspective. The findings underscore the importance of perioperative dialogue in helping patients feel safe and supported in vulnerable and unfamiliar care situations.

The present study has several limitations that should be taken into account when interpreting its findings. First, the study only involved NAs from three different settings, which means that the sample may not be representative of all NAs in Sweden or other healthcare systems. Second, the study was conducted over a relatively brief period, which may not capture the full complexity of the perioperative dialogue's use and impact over time. In addition, the data were collected from a small number of NAs who were specifically recruited by the author, further limiting the ability to generalize the findings. Consequently, while the study provides valuable insights into the experiences of NAs within a specific surgical department, the results cannot be extended to broader contexts or populations without further research.

## Conclusions

The perioperative meetings need to be developed and structured to enhance communication. A method for evaluating the outcomes of these conversations should be established. Additional recommendations include improving patient involvement in the dialogue and developing NAs' skills to enhance the quality of these interactions. The continuity provided by perioperative dialogue likely impacts both patient satisfaction and safety. Providing better patient information, offering it in multiple languages, and making efforts to maximize the benefits while eliminating disadvantages in perioperative meetings are also suggested. Further research would be valuable to clarify the importance of perioperative dialogue as a guiding principle for both staff and patients.
